# The relation between vaccination status and referral to a consultation–liaison psychiatry service for hospitalized patients with COVID-19

**DOI:** 10.1186/s13030-023-00296-z

**Published:** 2023-11-15

**Authors:** Tomoe Nishihara, Mao Shibata, Ayako Ohashi, Kazutoshi Hiyama, Takafumi Yamashita, Mika Kuroiwa, Nobuyuki Sudo

**Affiliations:** 1 Department of Psychosomatic Medicine, National Hospital Organization Fukuoka Higashi Medical Center, Fukuoka, Japan; 2 Department of Psychosomatic Medicine, Graduate School of Medical Sciences, Kyushu University, Fukuoka, Japan; 3 Clinical Education Center, Kyushu University Hospital, Fukuoka, Japan; 4 Department of Epidemiology and Public Health, Graduate School of Medical Sciences, Kyushu University, Fukuoka, Japan; 5 Department of Psychiatry, National Hospital Organization Fukuoka Higashi Medical Center, Fukuoka, Japan; 6 Department of Neuropsychiatry, Graduate School of Medical Sciences, Kyushu University, Fukuoka, Japan; 7 Department of Infectious Disease, National Hospital Organization Fukuoka Higashi Medical Center, Fukuoka, Japan; 8 Department of Respiratory Medicine, National Hospital Organization Fukuoka Higashi Medical Center, Fukuoka, Japan; 9 Clinical Research Center, National Hospital Organization Fukuoka Higashi Medical Center, Fukuoka, Japan

**Keywords:** COVID-19, Consultation–liaison psychiatry service, Vaccination

## Abstract

**Background:**

Previous studies have shown that patients with Coronavirus Disease 2019 (COVID-19) are likely to be affected by delirium and other psychiatric complications. We aimed to evaluate the relation between COVID-19 vaccination status and referral of patients hospitalized with COVID-19 for consultation–liaison psychiatry services.

**Method:**

From the medical records used for this retrospective, single hospital-based study, 576 patients were identified who were over 18 years-of-age and hospitalized with a diagnosis of COVID-19 between March 2020 and March 2022. The data of 531 for whom the vaccine history was obtained from the medical records were available for analysis: 455 without and 76 with referral to consultation–liaison psychiatry. A history of COVID-19 vaccination at least two times was used in the analysis of the odds for referral to liaison psychiatric consultation: 95% confidence interval (CI) in multivariable logistic regression. The adjustment factors included sex, age, body mass index (BMI), severity of COVID-19, C-reactive protein level, medical history, and therapeutic factors such as the use of remdesivir, steroids, or mechanical ventilation.

**Results:**

The prevalence of psychiatric consultation was 14.3%. Patients without vaccination had a 7-times greater OR (95%CI:2.08–23.58) than vaccinated patients for a referral for consultation–liaison psychiatry services after adjusting for confounding factors.

**Conclusion:**

Non-vaccination was associated with a greater likelihood of referral for consultation–liaison psychiatry service among these hospitalized Japanese patients with COVID-19, even after adjusting for clinical and therapeutic factors. It is possible that vaccination greatly lessens the need for the referral of COVID-19 patients for consultation–liaison psychiatry services.

**Supplementary Information:**

The online version contains supplementary material available at 10.1186/s13030-023-00296-z.

## Background

It has been reported that patients with Coronavirus Disease 2019 (COVID-19) are likely to present with psychiatric symptoms. Possible mechanisms include direct viral actions on the central nervous system (CNS), systemic inflammation caused by cytokine storms and/or therapeutic drugs, isolation, and social stress [1, 2]. It has been reported that patients with COVID-19 who are critically ill or in an intensive care unit (ICU) are more likely than others to develop delirium and psychiatric symptoms, suggesting the influence of systemic inflammation, hypoxia, and therapeutic drugs [3, 4]. In a multicenter retrospective survey conducted as a project of the Japanese Ministry of Health, Labor, and Welfare on psychiatric symptoms among COVID-19 patients admitted to a designated medical institution for infectious diseases, the most common psychiatric symptoms were insomnia, confusion, and anxiety. Patients with delirium and stress disorder/adjustment disorder as the diagnosis accounted for majority of the cases (45 and 27%, respectively). In addition, delirium significantly increases with the severity of the illness [5].

In our hospital, patients hospitalized for COVID-19 were often referred to consultation–liaison psychiatry services performed by psychosomatic medicine doctors because of the presence of psychiatric complications (Table 1). However, as the vaccination rate increased the number of referrals to our consultation–liaison services rapidly decreased. Earlier reports have shown that COVID-19 vaccination has a preventive effect against COVID-19 and reduces the severity of the disease [6]. However, to date, it has not been clarified whether or not vaccination is associated with a reduction in psychiatric complications associated with COVID-19.

**Table 1:** Baseline characteristics according to COVID-19 vaccination status

	COVID-19 vaccination		*p* -value
	No (*n* = 422)	Yes (*n* = 109)	
Sex, male (%)	60.9	53.2	0.15
Age (years)^a^	60.9 ± 1.0	71.3 ± 1.9	0.01
BMI (kg/m^2^)^a^	23.7 ± 0.2	22.1 ± 0.5	0.01
CRP (mg/dl)^a^	8.11 ± 0.38	5.56 ± 0.65	0.01
Past history (%)			
Diabetes mellitus	27.3	21.1	0.19
Hypertension	36.3	44.0	0.14
Dyslipidemia	23.0	22.9	0.99
Chronic kidney disease	13.3	15.6	0.53
Heart failure	3.3	10.1	< 0.01
Malignancy	5.2	11.0	0.03
Cerebral disease ^b^	6.6	9.2	0.36
Dementia	8.5	10.1	0.61
Psychiatric diseases ^c^	4.3	3.7	1.00
Severity of COVID-19			< 0.01
Mild (%)	48.6	62.4	
Moderate (%)	40.5	35.8	
Severe (%)	10.9	1.8	
COVID-19 interventions (%)			
Remdesivir	41.0	45.0	0.46
Steroid	52.4	31.2	< 0.01
Mechanical ventilation	12.6	1.8	< 0.01

Data are presented as the number of subjects in each group, with percentages

^a^Data are shown as mean ± standard error (SE)

^b^Cerebrovascular disease, cerebral palsy such as Parkinson’s disease

^c^Psychiatric disease includes mood disorder (*n* = 6), seizure disorder (*n* = 4), anxiety disorder (*n* = 3), intellectual disabilities (*n* = 3) adjustment disorder (*n* = 3), and others (*n* = 3)

**Fig. 1:**
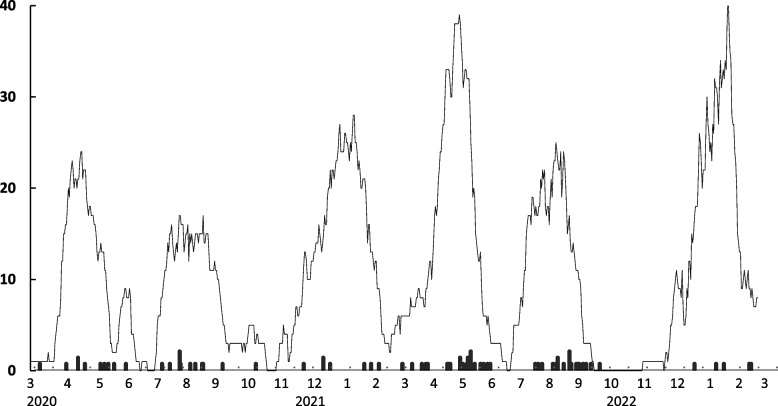
The flow of the number of COVID-19 patients admitted to the Covid-unit of Fukuoka Higashi Hospital. The black bar indicates the number of patients who were referred for consultation–liaison psychiatry service

The purpose of the current study was to determine if vaccination status is related to referral to consultation–liaison psychiatry service among Japanese patients hospitalized for COVID-19 at a designated infectious disease hospital. We also studied the influence of systemic inflammatory indices, such as severity and C-reactive protein (CRP), and treatment regimen, including steroids and mechanical ventilation management Fig 1.

## Materials and methods

### Subjects

**Fig. 2:**
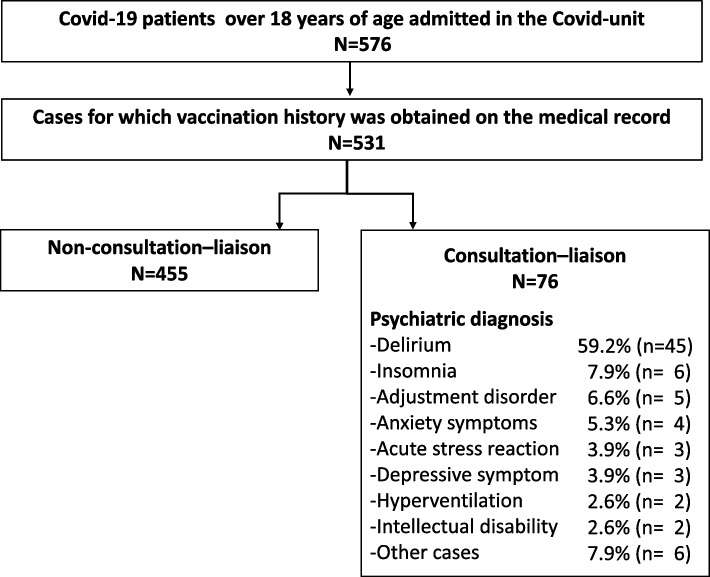
Study flow chart. The number of patients included in the study and those who were referred for consultation–liaison psychiatry service. The psychiatric diagnoses of patients were delirium, insomnia, adjustment disorder, anxiety symptoms, acute stress reaction, and depression

This study was a retrospective review of the medical records of a series of 576 COVID-19 patients aged 18 years or older who were treated as inpatients at the Fukuoka Higashi Medical Center from March 2020 to March 2022: 79 were referred for consultation–liaison psychiatry and 497 were not. Fukuoka Higashi Medical Center is a designated hospital for infectious diseases located near Fukuoka-City, Japan that has been treating COVID-19 patients since March 2020. Fig. 2 shows the number of patients studied and the number of consultations along with the psychiatric diagnoses. Vaccination history was confirmed from the medical records of 531 patients, but for 45 (7.8%), the information could not be confirmed and was treated as missing data.

### Criteria for referral for consultation–liaison psychiatry service

We defined consultation cases as patients who had a record of treatment by a psychosomatic specialist at the request of the attending physician. Our original criteria for referral for consultation–liaison psychiatry service are as follows:1) frequent psychiatric symptoms that require the attention of multiple staff members; 2) difficulty providing COVID-19 treatment unless psychiatric symptoms are promptly controlled; 3) difficulty managing the ward due to patients wandering around or frequently calling for nurses; 4) violent behavior that could endanger the medical staff; 5) when strong psychological symptoms such as hyperventilation or suicide attempts are present and professional mental care is needed; 6) when the patient requests more specialized mental health care; and 7) when the patient has a history of mental illness and is on medication.

### Assessment items

#### Vaccination history

Vaccination history was defined as two or more doses of a COVID-19 vaccine for which adequate vaccine efficacy has been reported in previous studies [6–8]. In Japan, two doses of the vaccine became available in March 2021, with government support: priority vaccination for healthcare workers began in March, for the elderly in April, and universal vaccination for persons under 60 years of age began in July. By August, 40% of the population had completed the two-dose vaccination regimen and 76% had by November. Messenger RNA-based vaccines by Pfizer/BioNTech and Takeda/Moderna were used for public vaccination. The vaccination history was investigated retrospectively by referring to medical records.

#### Potential confounding factors

Sex, age, and medical history, including psychiatric disorders, were by interview. For medical history, hypertension, diabetes mellitus, dyslipidemia, chronic kidney disease (CKD), heart failure, malignancy, cerebral disease, dementia, and psychiatric disease were extracted from the medical records. The body mass index (BMI) was calculated as weight (kg)/height (m2). We used a fully automatic system to measure the height and body weight. The weight of patients who could not stand was assessed using a suspension mechanism. The level of C-reactive protein (CRP) was measured by a quantitative method and used as a marker of inflammation: less than 0.3 mg/L was considered normal.

The classification of COVID-19 severity was based on the definitions in the guidelines of the Ministry of Health, Labour, and Welfare and the National Institute of Infectious Diseases, with mild disease defined as asymptomatic to coughing only with no shortness of breath at SpO2 ≥ 96%, moderate disease defined as shortness of breath or pneumonia findings at SpO2 < 96%, and severe disease defined as admission to the ICU or mechanical ventilation management [9].

Therapeutic factors included the use of remdesivir, steroids, other drugs, and mechanical ventilatory management. Based on the guidelines, COVID-19 drugs, including remdesivir (200 mg for the first dose and 100 mg for the following 10 days), were administered to patients with moderate or severe disease. Steroids for systemic inflammation were started with 6.6 mg/day of dexamethasone, then tapered: 1000 mg of methylprednisolone was added for three days in severe cases. They were used when respiratory failure was observed. In the case severe disease, continuous intravenous dexmedetomidine, propofol, and fentanyl are used for sedation during mechanical ventilation management.

### Statistical analyses

All continuous variables are expressed as the mean ± standard error (SEM). All analyses were performed using the SPSS version 22.0 J statistical software package (IBM SPSS Statistics, Chicago, IL, USA). The Mann–Whitney U test was used to evaluate differences in age, BMI, and CRP level. The X2 test was used to analyze nominal data. Odds ratios (OR) with 95% confidence intervals (CI) were calculated with logistic regression analysis adjusted for sex, age, and potential confounding factors that were chosen from clinical parameters, medical histories, and therapeutic factors using a backward method. *p* < 0.05.

## Results

The overall consultation rate was 14.3% (76/531; 73 in the non-vaccination group and 3 in the vaccination group). The psychiatric diagnoses of consultation cases included delirium (59.2%, *n* = 45), insomnia (7.9%, *n* = 6), adjustment disorder (6.6%, *n* = 5), anxiety symptoms (5.3%, *n* = 4), acute stress reaction (3.9%, *n* = 3), depression (3.9%, *n* = 3), hyperventilation syndrome (2.6%, *n* = 2), intellectual disability (2.6%, n = 2), and one each with a medical history of schizophrenia, bipolar disorder, generalized anxiety disorder, panic disorder, somatoform disorder, or neurodevelopmental disorder) (Fig. 2).

Table 1 shows the characteristics of the patients in the groups with and without vaccination. The vaccinated group was significantly older and had a higher BMI than the non-vaccinated group. The vaccinated group had lower levels of severity and CRP, a higher medical history of heart failure or malignancy, and significantly less use of steroids and ventilators.

**Fig. 3:**
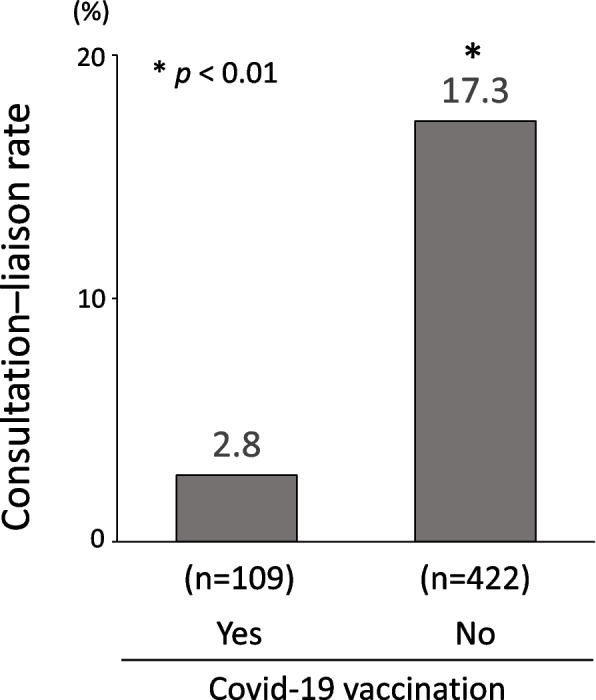
Difference in consultation–liaison rate by vaccination history. The vaccination group had a significantly lower consultation rate than the non-vaccination group

The vaccinated group had a significantly lower consultation rate than the non-vaccinated group (*p* < 0.01) (Fig. 3). Table 2 shows the adjusted odds ratios for referral of patients with COVID-19 for consultation according to the history of vaccination. Patients without COVID-19 vaccination were associated with a greater likelihood of consultation after adjustment for sex and age (Model 1) (adjusted OR = 7.78, 95% CI:2.40–25.24, *p* = 0.001). After adjusting for the covariates in Model 1 plus clinical parameters (BMI, the severity of COVID-19, CRP level, and history of heart failure, malignancy, and psychiatric diseases) (Model 2), the associations remained significant (adjusted OR = 7.54, 95% CI:2.26–25.22, *p* = 0.001). Moreover, after further adjustment for therapeutic factors (the use of remdesivir, steroid, and mechanical ventilation) (Model 3), a significant association remained (adjusted OR = 7.00, 95%CI:2.08–23.58, *p* = 0.002). Lastly, we added the adjustment for covariates (sex, BMI, history of psychiatric disease, use of steroids, and mechanical ventilation) that were chosen by backward elimination in Model 3. The significant association remained after adjustment by the forced entry method (adjusted OR = 7.19, 95%CI:2.14–24.17, *p* = 0.001). We also assessed the data after excluding patients with a history of psychiatric illness (supplementary material). The finding that non-vaccination resulted in a significantly greater likelihood of consultation was not substantially altered (adjusted OR = 5.16, 95%CI:1.54–17.31, *p* = 0.008).

**Table 2:** Odds ratios and 95% confidence intervals for referral for consultation–liaison psychiatry service, by history of COVID-19 Vaccination adjusted for factors in four models

COVID-19 vaccination	Number of consultation–liaison cases / patients	Model 1	Model 2	Model 3	Model 4
		(Adjusted for sex and age)	(Model 1 + clinical parameters)	(Model 2 + therapeutic factors)	(Adjusted for factors significant in Model3)
		aOR (95%CI)	aOR (95%CI)	aOR (95% CI)	aOR (95% CI)
Yes	3 / 109	1.00 (Reference)	1.00 (Reference)	1.00 (Reference)	1.00 (Reference)
No	73 / 422	7.78 (2.40–25.24)	7.54 (2.26–25.22)	7.00 (2.08–23.58)	7.19 (2.14–24.17)
*p* value		0.001	0.001	0.002	0.001

Model 1: Adjusted for sex and age

Model 2: Adjusted for covariates included in Model 1 + clinical parameters (BMI, COVID-19 severity, CRP level, and history of heart failure, malignancy or psychiatric disease)

Model 3: Adjusted for covariates included in Model 2 + therapeutic factors (use of remdesivir, steroid, and mechanical ventilation)

Model 4: Adjusted for covariates (sex, BMI, history of psychiatric disease, use of steroid and mechanical ventilation) that were chosen by backward elimination of the covariates in Model 3

## Discussion

Data from this hospital-based, retrospective study indicate that not being vaccinated for COVID-19 was associated with a greater likelihood of referral to consultation–liaison psychiatry services among patients hospitalized with COVID-19, even after covariates such as clinical parameters and therapeutic factors were included in the analysis. This finding indicates that COVID-19 vaccination reduces the likelihood that patients with COVID-19 will develop psychiatric complications.

### COVID-19 and psychiatric complications

In this study, 16.7% of all patients ≥18 years-of-age who were hospitalized with COVID-19 and for whom a vaccination history was obtained were referred for liaison psychiatric consultation, more than half of whom were diagnosed with delirium, insomnia, anxiety symptoms, and stress reactions.

It has been reported that COVID-19 patients have a high-prevalence of psychiatric complaints. One study reported that up to 80% of hospitalized patients with COVID-19 presented with neurological symptoms [10]. Since the beginning of the COVID-19 epidemic, insomnia, PTSD, depression, anxiety, and obsessive-compulsive symptoms have been reported to be highly prevalent [4, 11]. In addition, altered cognition has been seen in patients hospitalized with severe illness and in others without respiratory failure [3, 12]. Some brain imaging studies have reported neuroradiological patterns in patients with COVID-19, such as microhemorrhage, ischemic vascular damage, and encephalitis [13, 14]. In contrast, in other studies of patients with significant psychiatric symptoms no abnormal imaging findings were detected. The underlying mechanism of the psychoneurotic effects of coronaviruses may be direct or indirect. Previous studies have suggested pathological findings of SARS-CoV-1 in the cytoplasm of cortical and hypothalamic neurons to be a direct mechanism [15]. In addition to direct CNS entry via the vulnerable blood-brain barrier, SARS-CoV-2 may travel to the brain through direct CNS penetration [16]. Another potential molecular mechanism is the hijacking of mitochondrial bioenergetics [17]. Indirect mechanisms include the effects of hypoxia and cytokine storms on the central nervous system [18, 19].

In a previous larger multicenter survey of consultation–liaison psychiatry services at hospitals designated for infectious diseases in Japan, delirium and stress disorder/adjustment disorders (45 and 27%, respectively) accounted for the majority of mental symptoms exhibited by patients [5]. Delirium was more common in our study than in the previous study, although the proportion of severely ill patients needing psychiatric consultation was larger in the previous study (37.6%, this study 21.1%). One possible reason for this might be the particularly high rate of steroid use in our hospital (75.6%, *n* = 208/275). In addition, almost all patients on mechanical ventilators received a combination of opioids and sedatives.

Among the consultation cases, diagnoses other than delirium included insomnia, adjustment disorders, anxiety, acute stress reactions, and depression. The diagnoses in the present and previous studies are consistent with the categories of stress-related reactions and psychological distress. Differences in the descriptive diagnoses may be due to differences in the evaluators, time elapsed since the COVID-19 epidemic began, and/or social differences due to differences in the care environment and country of origin.

### Effect of vaccination on COVID-19

COVID-19 vaccination is known to reduce the chance of contracting the disease, hospitalization, and ventilator use 7–14 days after the second vaccination [6–8]. The present study also showed a significant reduction in severity, inflammation, and the use of steroids and ventilators, consistent with the results of previous studies. In addition, the current study showed that vaccination was related to fewer referrals for consultation–liaison psychiatry services, suggesting that vaccination reduces severe psychiatric complications. Possible mechanisms for this are the decrease in systemic inflammation and the concomitant decrease in the administration of drugs with psychiatric side effects, such as steroids and opioids; however, even after adjusting for these factors, non-vaccination was associated with an increased risk of referral in the present study. It is possible that the direct effects of coronaviruses on the CNS are suppressed by vaccination.

### Limitations

The present study has some limitations. First, the number of vaccinated patients may not be completely accurate because we had missing data for a small number of patients (7.8%, *n* = 45) due to a lack of information in the medical records. Further prospective studies will be necessary to obtain a more accurate analysis. In addition, information on the virus strain was not available for the majority of patients; therefore, we were unable to adjust for possible differences in the strains. Our hospital has set definite criteria for when physicians should consider referral for consultation–liaison psychiatry service, but the recommendation for consultation may have bias because it depends on the attending physician’s judgment. However, there has been no change in the attending physicians in the Department of Infectious Diseases since before the start of vaccination at our hospital, thus, we consider the impact on the number of consultations to be minimal.

## Conclusion

Our findings show that not being vaccinated for COVID-19 was associated with a greater likelihood of referral for consultation–liaison psychiatry service among this group of Japanese patients hospitalized for the treatment of COVID-19. It is possible that vaccination reduces the psychiatric complications of patients with COVID-19.

### Supplementary Information

**Additional file 1.**

## Data Availability

Data supporting the findings of this study have been included in this published article.
