# Supplementary material for: The relation between vaccination status and referral to a consultation–liaison psychiatry service for hospitalized patients with COVID-19
Source: Biopsychosoc Med. 2023 Nov 15;17:40. doi: 10.1186/s13030-023-00296-z (PMC10647145; doi:10.1186/s13030-023-00296-z)
Supplement: Supplementary file 1 — Additional file 1. [file 13030_2023_296_MOESM1_ESM.docx]

| Supplementary material  Odds ratios and 95% confidence intervals for referral for consultation–liaison psychiatry service by COVID-19 vaccination status adjusted for confounding factors when patients with a past history of a psychiatric disease were excluded. | | | | | |
| --- | --- | --- | --- | --- | --- |
| COVID-19 vaccination | Number of consultation–liaison cases / patients | Model 1 | Model 2 | Model 3 | Model 4 |
|  |  | (Adjusted for demographic factors) | (Model 1+clinical parameters) | (Model 2+therapeutic factors) | (Adjusted for factors  significant in Model3) |
|  |  | aOR (95%CI) | aOR (95%CI) | aOR (95 % CI) | aOR (95 % CI) |
| Yes | 1 / 109 | 1.00 (Reference) | 1.00 (Reference) | 1.00 (Reference) | 1.00 (Reference) |
| No | 64 / 422 | 7.15 (2.17-23.56) | 5.90 (1.77-19.60) | 5.16 (1.54-17.31) | 5.36 (1.60-17.94) |
| *p* value |  | 0.001 | 0.004 | 0.008 | 0.006 |
| Model 1: Adjusted for sex and age. | | | | | |
| Model 2: Adjusted for the covariates included in Model 1 + clinical parameters (BMI, COVID-19 severity, CRP level, and a history of heart failure, malignancy, or psychiatric disease). | | | | | |
| Model 3: Adjusted for covariates included in Model 2 + therapeutic factors (the use of remdesivir, steroid, and mechanical ventilation). | | | | | |
| Model 4: Adjusted for covariates (sexr, BMI, the history of psychiatric disease, the use of steroid and mechanical ventilation) that were chosen by backward elimination of the covariates in Model 3. | | | | | |
